# Supplementary material for: Complexome profiling of the Chlamydomonas psb28 mutant reveals TEF5 as an early PSII assembly factor
Source: Plant Cell. 2025 Mar 17;37(6):koaf055. doi: 10.1093/plcell/koaf055 (PMC12164590; doi:10.1093/plcell/koaf055)
Supplement: koaf055_Supplementary_Data [file koaf055_supplementary_data.zip › psb28_interactive.zip!/plots/intensity/qualityPlots/peptides/PSRP3_Cre02.g083950.t1.1.html]

### Cre02.g083950.t1.1

Trivial name: PSRP3  
SeqCoverage [median]: 17.10  
Mass: 33.37 kDa  
  
 

| Sequence | MissedCleavage | Mass [Da] | wtIntensity | muIntensity | Score |
| --- | --- | --- | --- | --- | --- |
| AVEDVMASK | 0 | 948.46 | 0.00 | 3961.41 | 101.72 |
| KDENIPLPEYR | 1 | 1372.70 | 0.00 | 479.27 | 36.30 |
| LAAGELSEAELSK | 0 | 1316.68 | 305.40 | 6910.31 | 167.12 |
| YAEAAVSSDDKALMEPLK | 1 | 1936.95 | 0.00 | 532.92 | 50.39 |
|  |  |  | 305.4 | 11883.9 |  |
